# Formula supplementation of breast-fed infants increases the incidence of cow’s milk protein allergy

**DOI:** 10.1186/2045-7022-5-S3-P88

**Published:** 2015-03-30

**Authors:** Gillian Dunn Galvin, Eimear Kelly, Audrey Dunn Galvin, Paul Murphy Brendan, Jonathan O B Hourihane

**Affiliations:** 1 University College Cork, Cork, Ireland

## Aim

One motivation for breast feeding is to prevent allergic disease in infants. Many breast fed babies in Ireland receive formula supplementation, within 24 hours of birth, though there are few medical indications for this. We explored (i) impact of upplementation on the likelihood of developing Cow's Milk Protein Allergy (CMPA), and (ii) current practice of formula supplementation (<24h) among mothers intending to breast feed.

## Method

55 CMPA-diagnosed children, with 3 feed type levels (breast only; formula only; breast with formula supplementation) and 55 non allergic, age and sex matched controls (born 2010-2011) were identified retrospectively in Cork University Maternity Hospital. Logistic regression (LoR) examined formula supplementation on likelihood of developing CMPA. Formula supplementation was prospectively measured among a separate group of 179 breast-feeding mothers. Linear regression (LiR) analysis was used to examine reasons and pre-existing factors for supplementation.

## Results

(i) LoR examined which feed type predicted development of CMPA. The model, controlling for parental atopy and infant sex, showed only formula supplementation was significant, (χ² (3) = 25.74, p < 0.05) with 74% diagnostic accuracy. Infants given top-up formula were 7.03 (95% CI, 1.82 - 27.25) times more likely to exhibit CMPA than those who were breast-fed only.

(ii) Frequency of current formula supplementation in breast fed infants (<24h) is 45.8%, with 56% administered by mother. LiR examined whether reasons for supplementation predicted development of CMPA. This model (incl. nurse advice and maternal/infant perceived well-being) was significant (F(8,170)=66.95, p<0.05) with 75% accuracy. In relation to pre-existing factors, only type of delivery predicted whether a mother would supplement with formula.

## Conclusion

Formula supplementation of breast fed babies in the first 24h is rarely medically indicated and is strongly associated with an increased risk of developing CMPA. The impact of professional advice is important. Attention should be given to measures aimed at reducing this potentially harmful practice.

